# Silent Circuit: Incidental Finding of Wolff-Parkinson-White Pattern in Late Pregnancy Complicated by Severe Preeclampsia

**DOI:** 10.7759/cureus.106616

**Published:** 2026-04-07

**Authors:** Srivane Richard, Dea Thomas, Usaid Raqeeb, Asher Gorantla, Suzette Graham-Hill

**Affiliations:** 1 Internal Medicine, State University of New York Downstate Health Sciences University, Brooklyn, USA; 2 Cardiology, State University of New York Downstate Health Sciences University, Brooklyn, USA

**Keywords:** electrophysiology, maternal arrhythmia, preeclampsia, pregnancy, supraventricular tachycardia, ventricular pre-excitation, wolf–parkinson–white pattern

## Abstract

Wolff-Parkinson-White (WPW) pattern is a rare conduction abnormality that may predispose to supraventricular tachyarrhythmias and sudden cardiac death. A 37-year-old gravida 2 para 1 woman at 35+6 weeks gestation, admitted with preterm labor and new-onset preeclampsia, developed transient chest tightness and dyspnea. Electrocardiography revealed sinus rhythm with short PR interval (the duration between the onset of atrial depolarization and the onset of ventricular depolarization), widened QRS complex (duration of ventricular depolarization), and delta wave consistent with ventricular preexcitation. Cardiac biomarkers and echocardiography were normal. She remained hemodynamically stable without sustained arrhythmia. As such, conservative telemetry monitoring was pursued. Her course progressed to severe preeclampsia requiring emergent cesarean delivery, which was uneventful. Postpartum Holter (14-day) monitoring redemonstrated delta waves with only one run of supraventricular tachycardia. She continued to remain asymptomatic at follow-up with referral for electrophysiologic evaluation. This case highlights the importance of recognizing incidental WPW during pregnancy and ensuring postpartum risk stratification for long-term management.

## Introduction

Wolff-Parkinson-White (WPW) pattern is a rare electrocardiographic finding caused by an accessory atrioventricular pathway that bypasses normal atrioventricular nodal conduction, resulting in premature ventricular activation and the characteristic short PR interval, widened QRS complex, and delta wave [1,2]. WPW syndrome is diagnosed when this pattern is accompanied by symptomatic tachyarrhythmias or related symptoms. While many patients remain asymptomatic, ventricular preexcitation can predispose to supraventricular tachyarrhythmias and, rarely, sudden cardiac death through rapid antegrade conduction over the accessory pathway during atrial fibrillation, producing preexcited atrial fibrillation that may degenerate into ventricular fibrillation [1-3]. Pregnancy introduces physiologic changes such as increased plasma volume, enhanced sympathetic tone, and hormonal modulation of myocardial conduction, which may increase susceptibility to arrhythmia in predisposed patients [3]. Current guidelines recommend conservative monitoring in asymptomatic, hemodynamically stable pregnant patients, with pharmacologic therapy reserved for documented sustained arrhythmias or hemodynamic compromise [3,4]. Incidental detection of WPW during pregnancy is uncommon, particularly when associated with obstetric complications such as preeclampsia. Here, a case of incidental WPW pattern was identified during late pregnancy in a patient admitted with preeclampsia who underwent emergent cesarean delivery.

## Case presentation

A 37-year-old African American gravida 2 para 1 woman at 35+6 weeks of gestation was admitted to obstetric services for preterm contractions and elevated blood pressure consistent with new-onset preeclampsia. During routine phlebotomy shortly after admission, she developed transient chest tightness and dyspnea lasting several minutes without palpitations, presyncope, or syncope.

At the time of symptom onset, her blood pressure was 148/92 mmHg, her heart rate was 88 beats/min, and her oxygen saturation was 98% on room air. Cardiovascular examination revealed normal heart sounds with regular rhythm and no murmur or friction rubs. Lungs were clear to auscultation bilaterally, and abdominal examination was significant for a gravid uterus in keeping with a 35-week gestation.

Her medical history was unremarkable for any known cardiovascular disease or previous arrhythmia diagnosis. She denied any history of prior palpitations, exertional chest pain, orthopnea, or exercise intolerance, although she described one isolated syncopal episode two years earlier that had not undergone formal evaluation. No family history of sudden cardiac death. She denied tobacco or alcohol use and reported only prenatal vitamin use during pregnancy.

**Figure 1:**
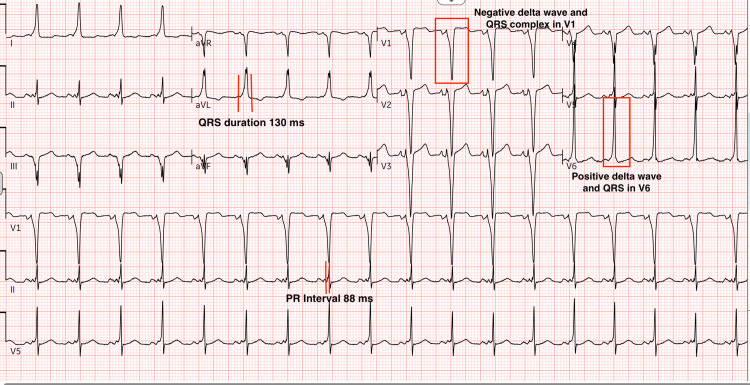
Electrocardiogram showing normal sinus rhythm, shortened PR interval, prolonged QRS duration with negative delta wave/QRS complex in V1 and positive delta wave/QRS complex in V6 consistent with WPW pattern type B WPW: Wolff-Parkinson-White.

**Figure 2:**
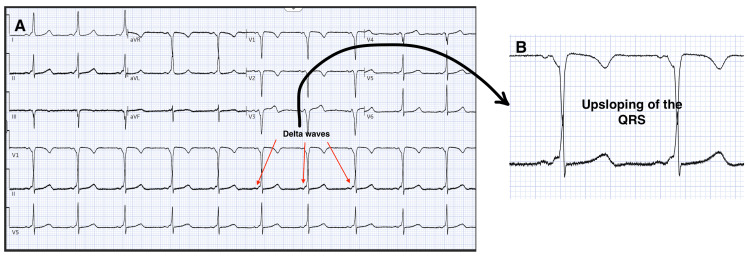
Electrocardiogram showing (A) delta waves, a specific feature of WPW pattern and (B) a magnified region highlighting the "up-sloping" morphology of the delta wave WPW: Wolff-Parkinson-White.

Initial workup with electrocardiography demonstrated sinus rhythm with a PR interval of 90 ms, QRS duration of 130 ms, and a delta wave consistent with ventricular preexcitation (Figures 1, 2).

Serum troponin I was negative. A complete blood count, a comprehensive metabolic panel, and a thyroid-stimulating hormone level were all within normal limits. Transthoracic echocardiography was performed, showing normal chamber dimensions, no valvular abnormalities, and preserved left ventricular systolic function with an ejection fraction of 60%.

She remained hemodynamically stable, and telemetry showed no sustained arrhythmia. Hence, conservative inpatient monitoring was pursued. Her hospital course progressed to severe preeclampsia requiring emergent cesarean delivery under epidural anesthesia. Delivery was completed without perioperative arrhythmia or hemodynamic instability, and both mother and neonate had favorable outcomes.

At six-week postpartum cardiology follow-up, a 14-day Holter monitoring redemonstrated delta waves and revealed four isolated supraventricular ectopic beats with one run of supraventricular tachycardia. There were no atrial fibrillation or ventricular arrhythmia recorded. Exercise stress electrocardiography with echocardiography was unremarkable and negative for ischemic changes. She remained asymptomatic and was referred for electrophysiology evaluation for further postpartum risk stratification.

## Discussion

This case highlights the important distinction between incidental ventricular preexcitation and clinically manifest WPW syndrome during pregnancy. Although the electrocardiographic pattern of WPW is defined by a short PR interval, widened QRS complex, and delta wave, the presence of these findings alone does not establish WPW syndrome unless symptoms or documented tachyarrhythmia are present [1,2]. In asymptomatic adults, ventricular preexcitation is often discovered incidentally, yet the principal clinical concern remains the possibility of sudden cardiac death. The principal mechanism of sudden cardiac death is rapid antegrade conduction over the accessory pathway during atrial fibrillation, producing preexcited atrial fibrillation that may degenerate into ventricular fibrillation even in previously asymptomatic individuals [1,2]. Contemporary data suggest that the lifetime risk of sudden cardiac death in asymptomatic preexcitation remains low but clinically relevant, particularly when high-risk features such as prior syncope or persistent preexcitation are present [4].

Pregnancy creates a unique proarrhythmic environment through increased plasma volume, elevated adrenergic tone, and hormonal influences on myocardial conduction, potentially facilitating atrioventricular reentrant tachycardia even in previously asymptomatic patients [2]. However, clinically significant arrhythmia remains uncommon, and most patients with isolated preexcitation tolerate pregnancy without major cardiovascular events when sustained tachyarrhythmia is absent. Current European guidance supports conservative inpatient observation when maternal hemodynamics are stable and no sustained arrhythmia is documented, reserving pharmacologic therapy for acute supraventricular tachycardia or hemodynamic compromise [1,2].

In this patient, transient chest tightness and dyspnea occurred during phlebotomy without associated tachycardia, hypotension, or telemetry-confirmed arrhythmia, making vasovagal activation or transient autonomic fluctuation more likely than accessory pathway-mediated tachycardia. Nevertheless, her prior unexplained syncopal episode significantly influenced risk interpretation because syncope in patients with ventricular preexcitation is recognized as a higher risk feature warranting additional evaluation [4]. Recent expert recommendations emphasize that patients previously considered asymptomatic should undergo formal risk stratification when syncope or presyncope is present because these symptoms may represent undocumented intermittent supraventricular tachyarrhythmia [4,5]. 

Current management strategies increasingly emphasize noninvasive risk stratification before invasive electrophysiologic testing. Ambulatory monitoring, exercise stress testing, and serial electrocardiographic assessment help evaluate persistence of preexcitation and potential accessory pathway refractoriness [4]. Abrupt disappearance of preexcitation during exercise suggests a lower-risk accessory pathway, whereas persistent preexcitation at higher sinus rates may justify invasive electrophysiology testing [4,5]. In this patient, postpartum Holter monitoring demonstrated only isolated supraventricular ectopy, and exercise stress testing did not provoke arrhythmia, suggesting a low immediate arrhythmic burden but not excluding long-term risk, as persistent baseline preexcitation remained present.

Definitive management of WPW increasingly favors catheter ablation when clinically indicated because radiofrequency ablation offers high procedural success with low complication rates. Contemporary recommendations support ablation in symptomatic patients, those with documented supraventricular tachycardia, high-risk electrophysiologic features, or preexcited atrial fibrillation [1,4]. For asymptomatic individuals, invasive electrophysiologic study is considered reasonable when prior syncope, occupational risk, or persistent preexcitation raises concern for rapid accessory pathway conduction [4,5].

An important therapeutic principle is avoidance of isolated atrioventricular nodal blocking agents in preexcited atrial fibrillation because agents such as beta-blockers, calcium channel blockers, digoxin, and adenosine may paradoxically accelerate conduction through the accessory pathway and precipitate ventricular fibrillation when atrial fibrillation is present [6]. In stable preexcited atrial fibrillation, intravenous procainamide or ibutilide remains preferred, whereas synchronized cardioversion is recommended when hemodynamic instability occurs [6]. Although our patient did not develop atrial fibrillation or sustained supraventricular tachycardia, recognition of this principle remains essential in obstetric settings where acute rhythm disturbances may arise unexpectedly during labor or delivery.

The coexistence of severe preeclampsia further increased cardiovascular complexity in this case. Hypertensive disorders of pregnancy are associated with endothelial dysfunction, sympathetic activation, and increased myocardial oxygen demand, factors that may theoretically lower arrhythmic threshold [7]. Epidural anesthesia likely contributed to perioperative hemodynamic stability by limiting sympathetic surges during cesarean delivery.

Postpartum electrophysiologic referral remained an important component of care because pregnancy-associated hemodynamic changes may transiently influence arrhythmic behavior, whereas definitive postpartum assessment allows evaluation of accessory pathway refractory characteristics without fetal procedural concerns. In this patient, an electrophysiology consultation was appropriate because prior syncope and persistent preexcitation placed her beyond a purely low-risk incidental category despite the absence of documented sustained arrhythmia during hospitalization. Early identification of the WPW pattern during pregnancy is crucial as recognition guides obstetric, anesthetic, and postpartum cardiac planning by identifying patients who may require rhythm surveillance and electrophysiologic risk stratification.

## Conclusions

Incidental WPW pattern identified during late pregnancy presents a unique clinical scenario requiring careful risk assessment. In hemodynamically stable patients without sustained tachyarrhythmia, conservative inpatient monitoring coupled with multidisciplinary obstetric planning can achieve favorable maternal and fetal outcomes. Postpartum evaluation, including ambulatory monitoring and electrophysiologic risk stratification, is essential, particularly in patients with prior unexplained syncope or persistent pre-excitation, to guide long-term management and consideration of definitive catheter ablation. This case highlights the importance of individualized care for incidental WPW in pregnancy and reinforces the role of proactive postpartum cardiovascular assessment.
